# The Impact of Social Media Shared Health Content on Protective Behavior against COVID-19

**DOI:** 10.3390/ijerph20031775

**Published:** 2023-01-18

**Authors:** Fathey Mohammed, Nabil Hasan Al-Kumaim, Ahmed Ibrahim Alzahrani, Yousef Fazea

**Affiliations:** 1 School of Computing, Universiti Utara Malaysia (UUM), Sintok 06010, Kedah, Malaysia; 2 Faculty of Technology Management and Technopreneurship, Universiti Teknikal Malaysia Melaka (UTeM), Durian Tunggal 76100, Melaka, Malaysia; 3 Computer Science Department, Community College, King Saud University, Riyadh 11437, Saudi Arabia; 4 Department of Computer & Information Technology, Marshall University, 1 John Marshall Drive, Huntington, WV 25755, USA

**Keywords:** protection motivation theory, perceived severity, social media fake news, e-health literacy, health experts participation, public awareness

## Abstract

The use of social media has increased during the COVID-19 pandemic because people are isolated and working from home. The use of social media enhances information exchange in society and may influence public protective behavior against the COVID-19 pandemic. The purpose of this study is to identify the factors affecting public protective behavior when relying on COVID-19 pandemic-related content shared on social media. A model based on Protection Motivation Theory (PMT) was proposed and validated using a quantitative survey approach. A questionnaire was distributed to random respondents, and 488 responses were received and analyzed using Smart-PLS software. The findings showed that perceived risk, e-health literacy, public awareness, and health experts’ participation influence public protective behavior when using social media to share COVID-19-relevant content. The outcomes of this study can enhance government agencies’ and public health care authorities’ understanding of how to use social media to raise awareness and reduce panic among the public.

## 1. Introduction

Social media facilitates the communication process and brings people together through content sharing. The platform enables individuals to search and discover accessible information that has become an indispensable source of information [1]. According to various definitions and terminologies, social media platforms include applications such as Facebook, blog, wiki, Flickr, YouTube, and Twitter [2]. Boyd and Ellison [3] defined social network sites as “web-based services that allow individuals to (1) construct a public or semi-public profile within a bounded system, (2) articulate a list of other users with whom they share a connection, and (3) view and traverse their list of connections and those made by others within the system. The nature and nomenclature of these connections may vary from site to site”. In addition, social media was introduced by Kaplan and Haenlein [4] as “a group of internet-based applications that build on the ideological and technological foundations of Web 2.0, and that allow the creation and exchange of user-generated content. Within this general definition, there are various types of social media that need to be distinguished further”. Activities including online video viewing, real-time chatting, commenting on each other’s content, reading and sending instant messages, creating, searching and sharing knowledge and information, and playing virtual games can be conducted over social media platforms [5]. Accordingly, during the crisis, social media became an important communication channel. During the COVID-19 pandemic, public health authorities worldwide and people moved to use social media channels to get connected and share information [6]. Moreover, social media offers individuals the ability to exchange instant disease-related information in real-time during the outbreak of COVID-19 [7]. According to [8], Twitter can deliver real-time content analysis to public health officials to provide the optimal response to public queries.

On the other hand, the spread of fake news tends to be a global issue [9]. The expansion usage of social media among the public during the pandemic lead to widening the space of concern about misleading news and information since the platform permits a quick interaction and dissemination of thoughts [10]. Social media users can promote ideas or spread the news by sharing, liking, or reposting; as a result, they are expected to be exposed to some sort of uncontrollable information, particularly news from independent social media users. Additionally, social media is proved to be an influential tool for disseminating large amounts of unfiltered content [11], leading to the phenomenon of misinformation. Fake news increases the difficulty of distinguishing authentic reports which should not be questioned [12]. According to Ruiz and Bell [13], health-related misleading and false information is often shared through social media. In this regard, as the world continues to explore cures for COVID-19, numerous pieces of research have indicated that the dissemination of fake news on social media has increased, and many experts believe this has exacerbated the threat of a pandemic [14]. The World Health Organization “WHO” stated that it is of serious concern to determine the main causes of fear, anxiety and anger, mainly through social media [15]. Several studies have demonstrated that the early signs of post-traumatic stress disorder (PTSD) may be exacerbated by incidental media exposure to mass trauma [15]. Misinformation and false news about COVID-19 raided social media during the outbreak time and fueled unfounded hysteria among many cybercitizens which in turns may generate confusion and hamper citizens’ mental well-being [16].

In addition, research has shown that the association between social media visits, personal risk perception, and protective behavior is resolved through fear and depression [7]. Some researchers have pointed out that these feelings affect the relationship between risk perception and mass media [7]. Moreover, during the COVID-19 pandemic, anxiety is seen by people primarily on social media as a major negative emotion [7]. When misleading or false information is passed on to the public, they appear to panic and react to such misinformation in many ways [17]. The rapid dissemination of incorrect information on social media, as well as users’ failure to detect accurate and misguided information, have increased community fears and concerns about the outbreak of COVID-19. Thus, social media should be utilized for smarter purposes since it provides an incentive to understand the steps to be taken and warn of false information and panic [18]. Accordingly, the effect of social media on individuals’ protective behavior can be influenced by different factors. Therefore, this study aims at investigating the elements that influence people’s protective behavior against COVID-19 based on social media shared content.

This paper is organized so that the related studies are presented in the next section. The development of the research model and the hypotheses is described next. Then, the applied research method to collect and analyze data to empirically validate the research model is presented. Next, the analysis of the data and the results are presented and then discussed. The research implications are described, and finally, the study is concluded.

## 2. Related Work

Social media may have a positive and negative impact on the individual’s attitude and behavior during emerging health threats. Liu, Fraustino [19] examined the impact of disaster information on the public’s disaster information-seeking and sharing as well as the likelihood of taking protective actions. Barman-Adhikari, Rice [20] assessed the impact of utilizing social media and HIV risk and protective behavior. Further, Chan, Winneg [21] studied the media effects on risk perceptions and behaviors concerning the Zika virus. Choi, Yoo [22] examined the role of social media exposure in shaping the public’s risk perceptions of the MERS outbreak.

In particular, social media has now been increasingly recognized as a critical factor shaping individuals’ attitudes regarding COVID-19 protective behaviors. Akdeniz, Kavakci [23] assessed the spread and frequency of protective behaviors and emotional and anxiety status among university students during the COVID-19 outbreak. In another context, Mat Dawi and Namazi [24] identified the perception of social media as a significant predictor of attitude toward COVID-19 preventive behavior. In addition, Mat Dawi, Namazi [25] examined the influence of social media on the public’s attitude toward adopting protective behavior.

El-Far Cardo, Kraus [26] identified the factors that influence people’s attitudes toward COVID-19 in Germany. This study’s results showed that trust in the health authorities and information about the virus from public media positively influence the public perception of the virus as a threat to health. On the other hand, reliance on social media for COVID-19 information negatively affects protection behavior. Fernández-Torres, Almansa-Martínez [27] studied the effect of the fake news spread on COVID-19 on the public’s perceptions in Spain. The results illustrated that false news related to COVID-19 has a high level of influence on public opinion. Alqahtani, Alrasheed [28] pointed out that trust in authorities, being anxious and worried, and levels of awareness are the most common factors affecting public perception to adopt protection practices during the COVID-19 pandemic.

Exploring the literature clearly indicates that the research related to investigating the impact of social media on the protective behavior of the public is growing, which was further echoed increasingly in using social media to share COVID-19-related information.

## 3. Theoretical Background and Hypotheses

The Protection Motivation Theory (PMT) provides a framework to understand individuals’ responses to fear appeals, including fear messages that encourage individuals to take protective actions [29]. It is mainly used to explain the impact of threat perception on changes in protection behaviors or attitudes. PMT suggests that when a threat message is exposed, two appraisals are initiated; threat appraisal and coping appraisal. The severity of the situation is assessed (threat appraisal), while coping appraisal determines how individuals can respond to the situation. The level of threat perception determines the acceptance or rejection of the message. Individuals’ evaluation of threats and coping appraisals influence their beliefs, attitudes, and behavioral intentions [30]. In the context of emerging health threats, threat appraisal may include perceived severity [31,32], perceived risks [31,33,34,35] and social media fake news [27,36]. On the other hand, a coping appraisal may include e-health literacy [35,37], public awareness [28,38] and scientist participation [39].

### 3.1. Perceived Severity

Severity refers to the significance of the threat, and perceived severity means the degree to which the person believes that threat has an impact on his health condition [40]. According to Kuang [31], perceived severity refers to the individuals’ beliefs about the magnitude and significance of a threat. The more serious people take a particular health problem, the more they try to reduce the risk of its occurring. In contrast, people who underestimate the risk of disease should act unhealthy. The perceived severity usually includes perceptions of the disease itself and its impact on the individual’s specific work and social roles [40]. Mya Kyaw, Aye [35] examined the perceived severity as a dimension of risk perception to COVID-19 among adults. In addition, Choi, Yoo [22] studied the impact of social media on the perceived severity of MERS. Accordingly, it can be hypothesized that:

**H1.**  *There is a significant relationship between COVID-19 perceived severity based on social media-shared content and public protective behavior*.

### 3.2. Perceived Risk

Perceived risk includes an individual’s subjective evaluation of the possibility of potential negative outcomes or diseases. According to Tyler and Cook [41], perceived risk can be classified into personal and social levels [42]. The former is suitable for calculating the serious impact of possible risks on the individual, while the latter involves raising the risk to society as a whole. Recent research shows that during a public health crisis, media can substantially influence people’s perception of risk issues [43]. As a digital medium for interpersonal communication, social media promotes the exchange of risk information. Studies show that compared with exposure to risk information, social media activities have a greater impact on individuals’ perceived risk [44]. The underlying explanation is that, compared with mass media, interpersonal communication is considered to be more collaborative, and individuals require more time in the psychological process of risk information [45]. Public reports through mainstream media that disseminate pandemic information and preventive measures throughout the pandemic may undermine the public’s understanding of risks. However, participating in social networking services may increase perceived risk in response to pandemic situations.

**H2.**  *There is a significant relationship between COVID-19 perceived risk based on social media-shared content and public protective behavior*.

### 3.3. Social Media Fake News

Fake news is identified as deliberately fabricated information that is shared to mislead and confuse people into believing lies or questionable verifiable facts [9]. According to Allcott and Gentzkow [46], fake news is described as any material that imitates real news reports but has inaccurate and misleading content. In the current study, the concept can be defined as untrue knowledge, including myths, misinformation, conspiracy theories, hoaxes, and misleading materials that are deliberately or unintentionally spread on social media sites [47] regarding COVID-19. Based on this concept, there is a difference between making and disseminating false content regarding COVID-19 on social networking sites. There is evidence that the use of social media sites has accelerated the flow of misleading online content [9]. From this perspective, we believe that if individuals avert a proper check, and this may be due to the millions of detailed information about COVID-19 on social media, misinformation may be spread. We argued that the tendency to validate messages prior to sharing is impossible at this moment of the pandemic, where everyone wants to be a reporter.

**H3.**  *There is a significant relationship between social media fake news on COVID-19 and public protective behavior*.

### 3.4. E-Health Literacy

The ability to scan, identify, understand, and evaluate health information from electronic sources and apply the acquired knowledge to solve health problems is described as e-health literacy [48]. The predictive factors of preventive measures are not only based on the external influence of social media but also include internal “assets”, including the collection of health information, skills, and abilities called health literacy. In terms of predictive health promotion and prevention, health literacy as a unique form of literacy has become increasingly important [49]. E-health literacy integrates and applies information and media literacy to e-health promotion. As people continue to seek medical advice from different online sources (particularly social media), e-health literacy is becoming more and more important. Previous research further illustrates that e-health literacy has a positive impact on health outcomes such as people’s health promotion habits during COVID-19 [37]. Mya Kyaw, Aye [35] recommend conducting awareness-raising activities and mass media health education focusing on protective behaviors. Thus, the following hypothesis is proposed:

**H4.**  *There is a significant relationship between e-health literacy based on social media-shared content and public protective behavior*.

### 3.5. Public Awareness

Public awareness is considered a key issue for directing the future of mankind. It includes multiple aspects of environmental conditions, such as the awareness, actions and attitudes of people toward a sustainable society. Social media data has a great impact on individuals and groups who connect to the online world and seek information for family, friends, and the public [50]. Previous research has identified that social media has caused panic among the public due to large amounts of data from unreliable sources [18]. The frequent circulating of information about the coronavirus outbreak continues to increase, and more data on its route of transmission are being collected around the world [51]. In a time when there is an absence of approved procedures to fight COVID-19, apart from no drug measures such as isolation and social distancing, individuals will continue to use and rely on social media, and it is necessary to mobilize the public and local communities to adopt isolation procedures.

**H5.**  *There is a significant relationship between public awareness based on social media-shared content and public protective behavior*.

### 3.6. Health Experts Participation

In order to share needs, resources, behaviors, and solutions, the COVID-19 emergency requires science and society to work together. For example, health experts ask individuals to implement real-time location tracking in their mobile phones to collect live data in order to be able to build accurate and reliable models of the spread of infectious diseases. At the same time, clinicians expect people to comply with the latest requirements of prevention and containment to respond effectively and to reduce the spread of the virus [52]. People further should trust health experts because they have experience related to technical and pragmatic expertise, capabilities, and resources to detect COVID-19 and provide accurate and effective drug therapies. Social media offers an opportunity for experts to quickly convey reliable information about hazards, and at the same time, others can have the opportunity to counter this by spreading misinformation. Therefore, the following hypnotized is concluded:

**H6.**  *There is a significant relationship between health experts’ participation in sharing information on social media and public protective behavior*.

## 4. The Study Research Model

**Figure 1:**
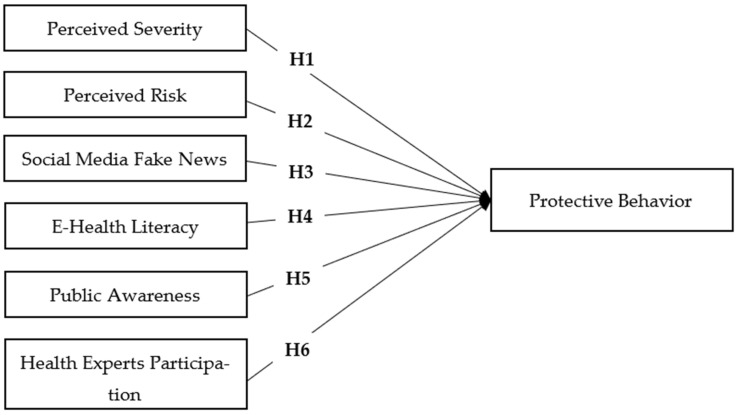
The Study Research Model.

Based on the theoretical background and by considering the context of using social media during COVID-19, a research model is proposed to identify the potential factors influencing protective behavior among the public. The proposed model holds a series of six independent determinants named perceived severity, perceived risk, social media fake news, e-health literacy, public awareness and health expert participation. The public’s protective behavior is the dependent variable that is affected by the other determinants. Table 1 presents the definitions and the concepts related to the model constructs based on the literature. Figure 1 represents the proposed model with the relationships and the hypotheses.

## 5. Research Methodology

The quantitative analysis examines the relationship between the study model determinants, which are measured and numerically analyzed using statistical techniques. Accordingly, this study employs a quantitative survey method to collect data from the targeted respondents. To ensure generalization, we apply probability sampling methods, and a Smart PLS3 is used to evaluate the data obtained from the survey.

### 5.1. Instrument Development

The instrument was developed to include twofold. Section A was designed to gather demographic information about respondents, such as gender, age, level of education and experience. The processing of this data enables researchers to understand the gaps between respondents in relation to certain attributes [54]. Section B was developed to examine the research model by analyzing and verifying the proposed hypotheses. To measure the constructs related to the factors, an ordinal scale is used. The measurements were extracted from the relevant literature and the elements adapted from different contexts, which were previously validated. However, to confirm the reliability of the instrument in the context of this study, a pilot test was conducted. Analyzing the data gathered from 34 respondents showed that the value of Cronbach’s alpha for all variables was acceptable, ranging from 0.683 (for perceived severity) to 0.833 (for protective behavior), which reflects good reliability and acceptable internal consistency of the measurements. Using a five-point Likert scale, respondents were asked to describe the degree to which they agreed or disagreed with each statement (1 = strongly disagreed; 2 = disagreed; 3 = neutral; 4 = agreed and 5 = strongly agreed).

### 5.2. Sampling Method and Data Collection

Probability random sampling was selected to avoid bias in the research and to ensure that samples are represented to the target population. An online survey was conducted, so a Google form was created to include the questionnaire’s items. The link to the Google form was distributed over social media. The study succeeded in collecting 506 questionnaires. But, a total of 18 questionnaires were considered defective responses and hence discarded since respondents lacked experience in using social media. As a result, 488 questionnaires were used for the analysis.

## 6. Analysis and Results

### 6.1. Respondent Profile

The descriptive statistics of the respondents for the current sample are listed in Table 2. The characteristics of the sample consist of gender, age, ethnicity, education level and monthly income/pocket money.

The number of male respondents was 264 (54.1%), and the number of females was 224 (45.9%). Most of the respondents belonged to the age group between 24–29 years old with a frequency of 209 (42.8%), while 93 respondents (19.1%) were 18–23 years old, 82 respondents were 30–35 years old (16.8%), and the rest were above 35 years old. Most of the respondents were at the SPM (high school) level of education, with a frequency of 153 (31.4%) and the Bachelor level, with a frequency of 123 (25.2%). Furthermore, analyzing the position of respondents, those employed full-time were on top with a frequency of 182 (37.3%), the number of those employed part-time was 124 (25.4%), and the rest were from other positions. Mostly the social media platforms most used to get news about the coronavirus disease is Instagram, with a frequency of 165 (33.8%) and Facebook, with a frequency of 106 (21.7%).

### 6.2. PLS Path Model Assessment

In the present study, structural equations utilizing Smart PLS 3.0 were modeled on a variance basis. Accordingly, the measurement model and structural model should be assessed. The measurement model uses the PLS Algorithm to evaluate the reliability and convergent and discriminating validity of the model, while the structural model addresses the assumption that the hypotheses are accepted or denied.

#### 6.2.1. Assessment of Measurement Model

Measurement model assessment includes measuring the internal consistency reliability, convergent validity and discriminatory validity. According to Hair Jr, Sarstedt [55], the measurement model stipulates evaluating the reliability (internal consistency reliability) and validity (convergent and discriminatory validity) of each construct in the model.

- Construct Reliability

Reliability is the extent to which the measurement scale is reliable in measuring the intended latent construct [56]. The reliability of a contract is established by testing for both consistency and stability of the scale measurement. Construct reliability can be evaluated using two criteria: Cronbach’s alpha and composite reliability. Cronbach’s alpha is a coefficient that indicates how well the items on a scale are positively correlated to each other. It is computed as the average intercorrelations among the items measuring the concept [57]. Construct reliability is achieved when Cronbach’s Alpha value is 0.7 or higher [58]. Due to Cronbach alpha’s limitations in the population, a different measure of internal consistency reliability can be more appropriately applied, which is known as composite reliability (CR) [55,59]. The construct reliability value of 0.70 or higher is acceptable [60,61]. Table 3 shows the construct reliability values for each construct. The reliability of all constructs is acceptable, with composite reliability values ranging from 0.841 to 0.921 and Cronbach’s Alpha values from 0.768 to 0.895.

- Construct Validity

Construct validity is the extent to which a construct measures what it is supposed to or aims to measure [62]. Construct validity measurement consists of convergent validity and discriminant validity [59,63]. Convergent validity, according to Hair Jr, Sarstedt [63], is the extent to which the measures of the same constructs are highly correlated, whereas discriminant validity is the level to which a construct is distinct from another construct.

Convergent validity determines the level of correlation of same-definition measures and addresses the loading and average variance extracted (AVE) of construction [63]. The higher outer loading of the indicator shows that the linked measurements have much in common or that items employed in testing the same definition harmonize with the construct [63]. Factor loadings exceeding 0.7 are deemed to be of great significance [64]. The loadings for all the items were greater than the recommended value of 0.7, as shown in Table. In addition, the value of average variance extracted (AVE) should be 0.5 or higher, which implies that constructs describe, on average, more than half of their indicator variance. In measurement items with AVE smaller than 0.5, there are, on average greater, errors. Table 4 shows the first outer loadings of the measurements and the AVE results.

On the other hand, discriminant validity suggests that measures of constructs that theoretically should not be related are not found to be highly correlated to each other [63]. According to [55], discriminant validity can be tested using the Fornell–Larcker criterion. The Fornell–Larcker criterion suggests that the square root of AVE values should be greater than the value of correlations with other constructs [63,65]. The square root of the AVE for each latent construct is more than its correlation with the other constructs (see Table 5).

#### 6.2.2. Assessment of Structural Model

The structural model is assessed after the measurement model, or the outer model is proven to be reliable and valid. Examining the model’s predictive abilities, as well as the relationships between the variables, would be part of the process [63]. Basically, the structural model is evaluated in order to assess the hypothesized relationships in the model. There are three parameters that determine the predictive abilities of the model, including the Coefficient of Determination (R^2^) of endogenous constructs, Effect Size (ƒ^2^) and Path Coefficients.

The amount of variance in dependent variables explained by the corresponding model is indicated by the R^2^ value. According to Hair Jr, Sarstedt [63], the R^2^ ranges from 0 to 1, with values of 0.75, 0.50, and 0.25 indicating significant, moderate, or weak predictive accuracy, respectively. The R^2^ value for endogenous construct (protective behavior) is 0.784. In addition to evaluating the R^2^ values of the endogenous construct, the effect size of the latent predictor construct was measured. The effect size (ƒ^2^) is used to determine whether the omitted construct has a significant impact on the endogenous constructs. As a rule of thumb, Cohen [66] described ƒ^2^ values of 0.02–0.14, 0.15–0.34, and greater than 0.35 as implying small, moderate and large effects, respectively. Table 6 below presents the ƒ² value for each path. The effect size ranges from a minimum value of 0.001 (SMFN → protective behavior) to a maximum value of 0.322 (EHL → protective behavior).

**Figure 2:**
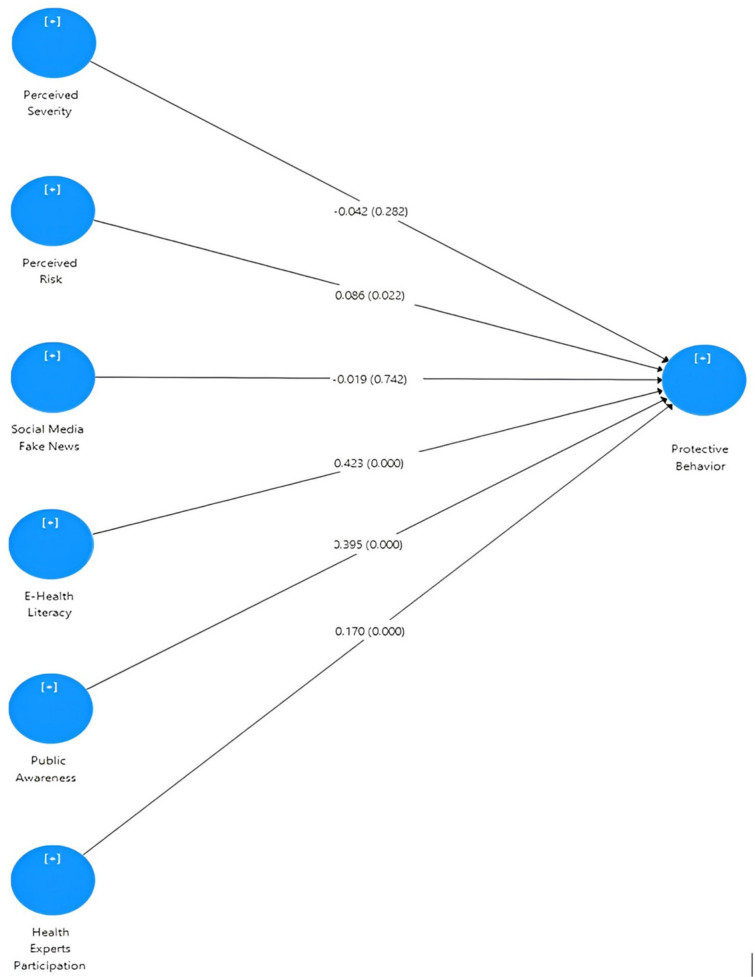
Structural Model for Path Coefficients and *p*-value.

Furthermore, the path coefficient is applied to determine the strength and significance of hypothesized relationships between latent constructs. Estimates are obtained for structural model relationships with standardized values ranging from −1 to +1, with coefficients closer to +1 indicating a strong positive relationship and coefficients closer to −1 indicating a strong negative relationship. The model’s path coefficients are shown in Figure 2. In order to test the significance of relationships, bootstrapping technique is used. The output reports the *t*-value path coefficients for all relationships. Table 7 presents the summary of the hypothesis testing results.

## 7. Discussion

This study proposed six main hypotheses to test the relationship between e-health literacy (EHL), health experts’ participation (HEP), perceived risk (PR), perceived severity (PS), public awareness (PA), and social media fake news (SMFN) as independent variables and protective behavior (PB) as the dependent variable. Table 7 shows the results for the structural model, which shows that path coefficients represent the hypnotized relationships.

The empirical results in this study indicated that the existence of a relationship between perceived severity and protective behavior in hypothesis H1 (PS → PB) of the structural model was rejected. The results (Table 7) indicate that the related *t* value was 1.110, which was less than the threshold value of *t* > 1.96. Accordingly, perceived severity was not supported as a factor affecting protective behavior when using social media-shared content during the COVID-19 pandemic. The results of this study contradict the studies in the literature [67,68,69]. However, the related results confirmed the findings in [70], which showed that perceived severity was not related to the intention to use a contact tracing app as a preventive behavior during the COVID-19 lockdown period. Also, Barakat and Kasemy [71] showed that the relationship between perceived severity and protective behavior was not significant in the context of COVID-19’s spread. This may be interpreted by considering the demographic data of the respondents in the current study. By referring to Table 2, more than 94% of the respondents were aged below 40 years, and their perception of COVID-19 severity was less. In addition, the results show that the *t* value associated with hypothesis H2 (PR → PB) was 2.397, indicating that perceived risk positively influences protective behavior. This was in line with the previous studies [43,72]. This may explain why during the occurrence of public health crises, as with COVID-19, social media had a substantial effect on people’s sense of risk issues and preventive actions.

Furthermore, the results indicated that the relationship between social media fake news and protective behavior, as in hypothesis (H3) in the structural model, was insignificant. The path coefficient for SMFN → PB was reported as −0.019, with a *t* value of 0.329, which was less than the threshold point of *t* > 1.96. Accordingly, social media fake news was not supported, as hypothesized, to affect protective behavior when using social media-shared content during the COVID-19 pandemic. This result contradicted the previous research results, which confirmed the existence of a relationship between social media fake news and individuals’ protective behavior [73,74]. The possible speculation to this result is that the high consideration from governments at higher levels worldwide, the applied long-term lockdown and the live updates regarding the situation by the social media portals and web pages of official authorities led to increasing awareness among the public. This also was confirmed in the results of hypotheses 4 and 5, which supported the impact of e-health literacy and public awareness on the protective behavior of the public towards COVID-19.

In addition, the results indicated that hypothesis H4 (EHL → PB) was accepted with a *t* value of 8.765. Hence, a positive relationship between e-health literacy and protective behavior was established. This is consistent with the results of [37], which confirmed the positive correlation between overall e-health literacy and infection-preventive behaviors among undergraduate students majoring in healthcare during the COVID-19 pandemic. The results also confirmed the findings of [35], which indicated that COVID-19 protective behavior was affected by the source of knowledge. This highlights the importance of the e-health literacy levels of the public. The official health authorities should design and develop educational programs and increase the level of e-health literacy and take further steps to direct suitable campaigns to deliver information to fight infectious diseases.

Hypothesis 5 (H5), which represents the relationship between public awareness and protective behavior, was supported based on the empirical results. Table 7 shows that the *t* value for the Path coefficient PA → CPB was 8.913, which establishes the significant influence of public awareness on protective behavior. This clearly indicates that public awareness was supported as a factor affecting protective behavior when using social media-shared content during the COVID-19 pandemic. Social networking sites, however, have become an effective tool for connectivity and the continuity of people’s habits in order to minimize physical contact and to become aware of or receive updates regarding the current situation. The current study results confirmed the view of previous studies [50,75] with regard to the influence of awareness during health crises on protective behavior among the public. Social media data has a significant influence on individuals and communities connecting to the online world and seeking information for family, friends and other general public.

Lastly, in relation to the relationship between health experts’ participation and protective behavior, hypothesis H6 (HEP → PB) also was supported with a *t* value of 3.658. This indicates that there is a significant relationship between health experts’ participation and protective behavior. The effect of COVID-19 and its media attention on the public’s protective behavior is growing individuals’ understanding of the role of experts in their lives. According to Ophir [76], experts can use social media to rapidly spread hazard information and help inform the public and patients on actions they can take to mitigate risk, which is aligned with the current study results. On the other hand, social media can rapidly spread misinformation across large sectors of the public; thus, social media should be considered by experts to understand and deploy effective communication strategies aimed at enhancing public protective behavior.

## 8. Conclusions

Since the declaration of COVID-19 as a pandemic, news media and social media have been deluged with information about the pandemic. Some of the information is intended to educate people about the newly emerged era of outbreaks, where management strategies were not yet ready or determined. Where the opposite side was true: misinformation about the pandemic and possible treatments was helping to alleviate people’s feelings of fear, anger, and panic. Protective behavior against COVID-19 is the subject of this study, which investigates the determinants that influence the impact of social media-shared content on public protective behavior. A model based on the Protection Motivation Theory (PMT) was proposed to identify and test the elected factors influencing public protective behavior by considering the impact of social media content. The study affirms that social media should be employed by government agencies and public health care authorities to raise awareness among the public and reduce panic among people. Globally governments should utilize social media to inform citizens about an impending hazards, thereby making the best use even with limited resources. The media should also be held accountable for the accuracy of their reports.

According to the research finding of this study, social media should be used to reinforce public health responses. The timely monitoring of public social media interactions during crises may promote awareness and help in developing the appropriate policies to mitigate risk and manage coping efficiently with the crisis. In addition, government authorities can use social media analytics to exchange and comment on real-time information about ongoing infectious disease threats. Furthermore, critical remedial measures against false information, as well as health interventions, should take place to prevent the collapse of health systems. Moreover, effective communication between official health authorities and the public is essential for the successful control of the pandemic. Social media can play a crucial role in transmitting updated policies and regulations from authorities to the public.

A toolkit should be developed to enhance public health response to face any possible future global outbreaks. It is particularly important when a lack of clear media communication strategies exists that require controlled transparency about the epidemic. In addition, the capacity of risk communication should be strengthened as an essential component of efforts to enhance national health security. Lastly, a real-time information-sharing system is required, which collects and analyses data from various social media platforms.

## Figures and Tables

**Table 1:** Factors affecting Protective Behavior against COVID-19.

Factors	Definition	Concepts	Author
Perceived Severity	A subjective calculation of the seriousness of a health condition and its possible implications.	The more seriously one takes a given health question, the more one attempts to reduce the risk of incidence of it.	(Rosenstock, Health and Winter, 1974) [40]
Perceived Risk	Involves the subjective examination of individuals as to the risk of potential negative outcomes or illnesses.	Interaction with social media has a greater impact on personal perceived risk than exposure to risk data.	(Xiaohua and Xigen 2017) [44]
Social Media Fake News	Fake news is knowledge intentionally invented to misinform and mislead people into believing lies or questionable verifiable evidence.	Social networking sites intensify the flow of fake web content.	(McGonagle, 2017) [9]
E-Health Literacy	The degree to which people have the ability to access, process and comprehend basic health information and resources required to make effective health decisions.	As people continue to seek medical advice from various web-based sources, particularly social media, it is important.	(Stawarz, Preist and Coyle, 1988) [49]
Public Awareness	It is a primary concern for the future of humanity.	It represents many aspects of environmental conditions, such as the awareness, actions and attitudes of people toward a sustainable society.	(Xu et al., 2013) [53]
Health Experts Participation	In order to share needs, resources, behavior and solutions, science and society work together.	Experts ask people to allow their real-time location tracking through apps and devices to build precise and accurate models of the spread of infection, making them the frontline data collector subjects.	(Remuzzi and Remuzzi, 2020) [52]

**Table 2:** Respondent Profile.

Items		Frequency	%
**Gender**	Male	264	54.1
	Female	224	45.9
	Total	488	100.0
**Age**	18–23 years old	93	19.1
	24–29 years old	209	42.8
	30–35 years old	82	16.8
	36–40 years old	75	15.4
	41 and above	28	5.7
	Total	488	100.0
**Level of Education**	High School	153	31.4
	Diploma	83	17
	Bachelor	123	25.2
	Master	62	12.7
	PHD	50	10.2
	Others	17	3.5
	Total	488	100.0
**Working Status**	Employed full time	182	37.3
	Employed part-time	124	25.4
	Student	97	19.9
	Unemployed	65	13.3
	Others	20	4.1
	Total	488	100.0
**The Social Media Platforms Most Used to Get News About the Coronavirus Disease**	Whatsapp	59	12.1
	Facebook	106	21.7
	Telegram	69	14.1
	Twitter	56	11.5
	Instagram	165	33.8
	Others	33	6.8
	Total	488	100.0

**Table 3:** Construct Reliability.

Construct	Cronbach’s Alpha	Composite Reliability
Protective Behaviour (PB)	0.807	0.866
E-Health Literacy (EHL)	0.768	0.841
Health Experts Participation (HEP)	0.802	0.856
Perceived Risk (PR)	0.894	0.921
Perceived Severity (PS)	0.895	0.918
Public Awareness (PA)	0.775	0.846
Social Media Fake News (SMFN)	0.818	0.853

**Table 4:** Convergent Validity Measurement Results.

Construct	Items	Loadings	Average Variance Extracted (AVE)
Perceived Severity	PS1	0.873	0.692
	PS2	0.906	
	PS3	0.810	
	PS4	0.821	
	PS5	0.742	
Perceived Risk	PR1	0.857	0.701
	PR2	0.858	
	PR3	0.813	
	PR4	0.859	
	PR5	0.797	
Social Media Fake News	SMFN1	0.894	0.546
	SMFN2	0.906	
	SMFN3	0.597	
	SMFN4	0.639	
	SMFN5	0.588	
E-Health Literacy	EHL1	0.724	0.513
	EHL2	0.746	
	EHL3	0.721	
	EHL4	0.682	
	EHL5	0.707	
Public Awareness	PA1	0.733	0.524
	PA2	0.706	
	PA3	0.702	
	PA4	0.737	
	PA5	0.741	
Health Experts Participation	HEP1	0.851	0.547
	HEP2	0.837	
	HEP3	0.690	
	HEP4	0.663	
	HEP5	0.628	
Protective Behavior	CPB1	0.822	0.566
	CPB2	0.814	
	CPB3	0.699	
	CPB4	0.708	
	CPB5	0.708	

**Table 5:** Fornell-Larcker Criterion.

Construct	PS	PR	SMFN	EHL	PA	HEP	PB
**PS**	**0.832**						
**PR**	0.822	**0.837**					
**SMFN**	0.584	0.540	**0.739**				
**EHL**	0.637	0.596	0.536	**0.717**			
**PA**	0.520	0.435	0.545	0.637	**0.724**		
**HEP**	0.794	0.716	0.447	0.624	0.614	**0.740**	
**PB**	0.480	0.460	0.734	0.605	0.637	0.390	**0.752**

Protective Behaviour (PB); E-Health Literacy (EHL); Health Experts Participation (HEP); Perceived Risk (PR); Perceived Severity (PS); Public Awareness (PA); Social Media Fake News (SMFN). The bold values refer to the square root of AVE.

**Table 6:** Result for Effect Size (ƒ^2^).

Path	*f* ^2^	Effect Size
EHL → Protective Behavior	0.322	moderate
HEP → Protective Behavior	0.054	low
PR → Protective Behavior	0.015	low
PS → Protective Behavior	0.003	low
PA → Protective Behavior	0.245	moderate
SMFN → Protective Behavior	0.001	low

**Table 7:** Table of Hypothesis Testing (Direct Effect).

Hypothesis	Path	Original Sample (O)	Standard Deviation (STDEV)	T Statistics (|O/STDEV|)	*p* Values	CI 2.5%	CI 97.5%	Results
**H1**	PS → PB	−0.042	0.038	1.110	0.268	−0.111	0.035	Rejected
**H2**	PR → PB	0.086	0.036	2.397	0.017	0.019	0.158	Accepted
**H3**	SMFN → PB	−0.019	0.057	0.329	0.742	−0.118	0.093	Rejected
**H4**	EHL → PB	0.423	0.048	8.765	0.000	0.324	0.511	Accepted
**H5**	PA → PB	0.395	0.044	8.913	0.000	0.304	0.477	Accepted
**H6**	HEP → PB	0.170	0.047	3.658	0.000	0.073	0.261	Accepted

## Data Availability

The data presented in this study are available on request from the corresponding author.
